# A Comprehensive Study of Risk Factors, Etiology, and Infarction Patterns in Cerebrovascular Accidents at a Tertiary Care Hospital in India

**DOI:** 10.7759/cureus.68433

**Published:** 2024-09-02

**Authors:** Raju Hansini Reddy, Pradnya Diggikar, Mayank Mundada, Arun Oommen, Tushar Pancholi, Bhavya Yammanuru, Sree Vidya Yekkaluru, Advit Sangwan

**Affiliations:** 1 General Medicine, Dr. D. Y. Patil Medical College, Hospital and Research Centre, Dr. D. Y. Patil Vidyapeeth (Deemed to Be University), Pune, IND

**Keywords:** ischemia, hemorrhagic stroke, toast criteria, risk factor, cerebrovascular accidents, stroke

## Abstract

Background

Stroke is a debilitating cerebrovascular condition characterized by sudden neurological deficits. The incidence of stroke is rising in India, posing significant public health concerns. This study aims to examine the risk factors and etiology of stroke using the Trial of Org 10172 in Acute Stroke Treatment (TOAST) classification and analyze infarct areas in cerebrovascular accidents (CVA) at a tertiary care hospital.

Methodology

This cross-sectional, hospital-based observational study was conducted at Dr. D. Y. Patil Medical College, Hospital and Research Centre, Dr. D. Y. Patil Vidyapeeth (Deemed to Be University), Pune, India, from January 2023 to January 2024. The study included 100 adult patients diagnosed with CVA based on clinical and radiological criteria. Patients aged 18 years and older were eligible, while those with a history of head trauma or those below 18 years were excluded. The investigation protocol included routine biochemical assessments and radiological investigations, such as computed tomography (CT), magnetic resonance imaging (MRI) with angiography or venography, and Doppler ultrasound of bilateral carotid arteries.

Results

The study population consisted of 100 patients, with 84 males (84%) and 16 females (16%). Age distribution showed 44% were over 60 years old, 23% aged 51-60 years, 15% aged 31-40 years, 14% aged 41-50 years, and 4% aged 21-30 years. Hypertension was the most prevalent risk factor, affecting 75% of patients, with a higher occurrence in males (62%), compared to females (13%). Smoking was observed in 51% of patients, and alcohol consumption was seen in 50%. Other significant risk factors included dyslipidemia (39%), diabetes mellitus (33%), chronic kidney disease (11%), ischemic heart disease (10%), atrial fibrillation (4%), valvular heart disease (4%), and pregnancy or postpartum conditions (2%).

Ischemic stroke was predominant, occurring in 80% of patients, while hemorrhagic stroke occurred in 20%. High occurrences of ischemic strokes were noted in the frontal lobe (41%), parietal lobe (37%), occipital lobe (27%), and temporal lobe (26%), with the internal capsule region also showing significant numbers (27%). According to the TOAST classification, the most prevalent cause of stroke in this study was undetermined etiology with two or more causes, accounting for 32% of cases, followed by large artery atherosclerosis, which accounted for 30%. Cardioembolic stroke was identified in 11% of the patients, with 4% due to atrial fibrillation, 3% due to acute myocardial infarction, 3% due to rheumatic valvular heart disease, and 1% due to infective endocarditis.

Conclusion

This study highlights the significant prevalence of hypertension, smoking, alcohol consumption, and hyperhomocysteinemia as major risk factors for stroke. Ischemic strokes were predominant, with high occurrences in the cerebral lobes and gangliocapsular region. These findings emphasize the need for targeted prevention strategies, including managing hypertension and lifestyle modifications such as smoking cessation and reducing alcohol consumption, to mitigate the risk of stroke. Effective management of blood pressure, lipid levels, and blood glucose is crucial for stroke prevention. Recognizing gender-specific differences and addressing comorbidities through an integrated approach can enhance patient outcomes and reduce the burden of stroke.

## Introduction

Stroke remains a major public health issue, with the rates of incidence, prevalence, disability-adjusted life years, and mortality related to stroke increasing significantly more in developing countries than in developed countries from 1970 to 2019. In 2019, stroke was the second-leading cause of death and disability globally [1]. The World Health Organization (WHO) defined stroke in 1970 as the rapid onset of clinical signs of global or focal cerebral dysfunction lasting for 24 hours or more or resulting in death due to a vascular cause. Recently, the American Stroke Association (ASA) expanded this definition to include any objective proof of permanent brain, spinal cord, or retinal cell death due to a vascular source, based on pathology or imaging results, regardless of clinical signs [2].

A cross-sectional study conducted at tertiary care hospitals in Nepal, Pakistan, India, and Eastern India revealed crucial insights into cerebrovascular accidents (CVA). The studies highlighted common risk factors such as hypertension, diabetes, smoking, and alcohol [3-7]. Ischemic strokes were more prevalent than hemorrhagic strokes, with hypertension being a significant risk factor for intracerebral bleeds [4]. The clinical profile of patients with posterior circulation ischemic stroke in Eastern India showed a male predominance, with diabetes, hypertension, dyslipidemia, and tobacco use as major risk factors, along with motor disturbances, vertigo, and gait ataxia as common manifestations [7]. These findings emphasize the importance of regular monitoring of blood pressure, blood sugar, and lipid profiles in individuals with risk factors to prevent the devastating impact of CVAs.

A case-control study published in *The Lancet*, conducted across 22 countries (INTERSTROKE), identified five primary risk factors responsible for over 80% of the total stroke risk: hypertension, abdominal obesity, diet, smoking, and exercise [8]. For ischemic stroke, significant associations were found with each of the nine risk factors identified in the INTERHEART study, collectively accounting for approximately 90% of the population-attributable risk (PAR). Data for hemorrhagic stroke, while based on fewer cases with wider 99% confidence intervals than for ischemic stroke, identified six important risk factors that contributed significantly to the PAR.

Identifying the etiologic subtypes of ischemic stroke is essential for managing individual patients, directing subtype-specific treatments, assessing public health trends, and mapping changing disease patterns across broad populations. The Trial of ORG 10172 in Acute Stroke Treatment (TOAST) classification system is the predominant method globally for categorizing ischemic stroke subtypes. TOAST classifies ischemic stroke into five subtypes: small vessel disease (SVD), large vessel atherosclerosis (LAA), stroke of other determined etiology (OTH), stroke of undetermined etiology (UND) with two or more causes, and cardioembolic stroke (CE). For patient-level analysis, the UND category is further subdivided into three groups: undetermined with more than one cause (UND-M), undetermined with incomplete work-up (UND-I), and undetermined despite complete work-up (UND-C) [9].

This study aims to examine the risk factors and etiology of stroke using the TOAST classification and to analyze infarct areas in CVA at a tertiary care hospital. By addressing these aspects, the study seeks to provide valuable insights into effective strategies for managing and preventing stroke in clinical settings.

## Materials and methods

Study design and setting

This cross-sectional hospital-based observational study was conducted at Dr. D. Y. Patil Medical College, Hospital and Research Centre, Dr. D. Y. Patil Vidyapeeth (Deemed to Be University), Pune, India, from January 2023 to January 2024. The study aimed to analyze risk factors, etiology, and infarction patterns in CVA. Before starting the study, approval was obtained from the Institutional Ethics Sub-Committee of Dr. D. Y. Patil Medical College, Pune (approval number: IESC/PGS/2022/05).

Inclusion criteria

All patients clinically and radiologically diagnosed with CVA and aged 18 years or older were included in the study.

Exclusion criteria

Patients below the age of 18 years and those with a history of head trauma were excluded from the study.

Sample size

Considering the proportion of CVA reported in previous studies, with a 95% confidence interval and an acceptance difference of 0.09%, the sample size calculated was 100 patients. This calculation was based on the study by Sheikh and Kulkarni using WinPepi software version 11.65 [10].

Data and sample collection

Patients were recruited from the medicine ward, intensive care unit (ICU), neurology, and emergency room of Dr. D. Y. Patil Medical College, Hospital, and Research Centre, Dr. D. Y. Patil Vidyapeeth (Deemed to Be University). Detailed clinical histories were taken from all patients, focusing on risk factors such as hypertension, smoking, alcohol consumption, diabetes mellitus, dyslipidemia, and prior strokes or transient ischemic attacks (TIA). Patients were examined for signs and symptoms of neurological deficits. The diagnosis of stroke was primarily based on clinical assessment and confirmed by neuroimaging, such as computed tomography (CT) or magnetic resonance imaging (MRI) of the brain, conducted within 72 hours of onset to determine the type of stroke and its vascular territory.

Blood samples were collected for various investigations, including complete blood count (CBC), erythrocyte sedimentation rate (ESR), fasting and postprandial blood sugar levels (BSL), arterial blood gases (ABG), D-dimer, vitamin B12, folic acid, prothrombin time-international normalized ratio (PT-INR), renal function test, liver function test, blood culture and sensitivity, electrocardiography (ECG), and 2D echocardiography. Additional tests such as antinuclear antibody (ANA) by immunofluorescence (IF), serum homocysteine levels, antiphospholipid antibodies (APLA), Protein C, and Protein S were performed where indicated. Lumbar puncture with cerebrospinal fluid (CSF) analysis was also conducted when necessary. Radiological investigations included CT brain, both standard and with angiography if indicated, performed using a 128-slice CT scanner. MRI brain, both standard and with angiography or venography, was conducted using a 3.0 Tesla MRI machine with sequences including T1-weighted, T2-weighted, fluid-attenuated inversion recovery (FLAIR), diffusion-weighted imaging (DWI), and MRI angiography/venography. Additionally, high-resolution B-mode ultrasound with color Doppler imaging was used for bilateral carotid arteries.

Consent

Written informed consent was obtained from all participants or their legally authorized representatives before inclusion in the study. Consent forms were translated into the participants' native languages to ensure they comprehended the study’s objectives and procedures. Participants were informed of their right to withdraw from the study at any time without any impact on their medical care.

Statistical analysis

Data were entered into a secure database and cleaned for missing or inconsistent entries. Preprocessing included the normalization of continuous variables and the categorization of qualitative data. Descriptive statistics (mean, median, and standard deviation) summarized patient demographics and clinical characteristics. Statistical analysis was performed using IBM SPSS Statistics for Windows, Version 25 (Released 2017; IBM Corp., Armonk, New York, United States) and R software version 4.0.3 (The R Foundation for Statistical Computing, Vienna, Austria).

## Results

Our study, involving 100 participants, provides a detailed demographic overview of the population. The sex distribution reveals a significant male predominance, with 84 (84%) of the patients being male and 16 (16%) female (Table 1). The age distribution shows that the largest proportion of patients, 44 (44%), are over 60 years old, followed by 23 (23%) in the 51-60 age range, and only four (4%) in the 21-30 age range. The mean age of the study population is 56 years, with a standard deviation of 14.42 years (Table 2). These tables illustrate that the study population is predominantly older and male, consistent with demographic trends commonly observed in cerebrovascular accident studies.

**Table 1:** Gender distribution among the study population n: number; %: percentage

Gender	n (%)
Male	84 (84%)
Female	16 (16%)
Total	100 (100%)

**Table 2:** Age distribution among the study population n: number; %: percentage; SD: standard deviation

Age group (years)	n (%)	Mean ± SD
21-30	4 (4%)	56 ± 14.42
31-40	15 (15%)	
41-50	14 (14%)	
51-60	23 (23%)	
>60	44 (44%)	

Among the risk factors for stroke, hypertension was the most prevalent, affecting 75 (75%) patients, with 62 (62%) males and 13 (13%) females. Smoking was observed in 51 (51%) patients, while alcohol consumption was reported in 50 (50%), predominantly among males. Dyslipidemia was present in 39 (39%) patients, diabetes mellitus in 33 (33%), and a history of stroke or TIA in 29 (29%). Other risk factors included chronic kidney disease in 11 (11%), ischemic heart disease in 10 (10%), atrial fibrillation in 4 (4%), valvular heart disease in 4 (4%), and a history of drug use in 4 (4%). Pregnancy or postpartum conditions were reported in 2 (2%) patients (Table 3).

**Table 3:** Distribution of risk factors among the study population TIA: transient ischemic attack; n: number; %: percentage

Risk factors	Female, n (%)	Male, n (%)	Total, n (%)
Hypertension	13 (13%)	62 (62%)	75 (75%)
Diabetes mellitus	5 (5%)	28 (28%)	33 (33%)
Dyslipidemia	4 (4%)	35 (35%)	39 (39%)
Chronic kidney disease	4 (4%)	7 (7%)	11 (11%)
Ischemic heart disease	1 (1%)	9 (9%)	10 (10%)
Atrial fibrillation	2 (2%)	2 (2%)	4 (4%)
Valvular heart disease	2 (2%)	2 (2%)	4 (4%)
Pregnancy/post-partum	2 (2%)	0 (0%)	2 (2%)
Alcohol consumption	2 (2%)	48 (48%)	50 (50%)
Smoking	6 (6%)	45 (45%)	51 (51%)
Drug history	2 (2%)	2 (2%)	4 (4%)
Past history of stroke/TIA	4 (4%)	25 (25%)	29 (29%)

Ischemic stroke occurred in 80 (80%) patients, while hemorrhagic stroke occurred in 20 (20%) patients (Table 4). The majority of strokes were ischemic, accounting for 80 (80%) cases.

**Table 4:** Distribution of ischemic and hemorrhagic strokes among the study population n: number; %: percentage

Stroke type	n (%)
Ischemic	80 (80%)
Hemorrhagic	20 (20%)
Total	100 (100%)

In our study, arterial strokes were overwhelmingly more common, accounting for 88 (88%) cases, while venous strokes represented 12 (12%) (Table 5).

**Table 5:** Arterial versus venous strokes n: number; %: percentage

Stroke type	n (%)
Arterial	88 (88%)
Venous	12 (12%)
Total	100 (100%)

This study details the incidence of ischemic, hemorrhagic, arterial, and venous strokes across various brain regions as seen in CT/MRI brain imaging. In our study, the cerebral lobes - frontal lobe (41 (14.49%)), parietal lobe (37 (13.07%)), both occipital lobe and internal capsule (27 (9.54%)), show high occurrences of ischemic strokes, followed by regions like the temporal lobe (26 (9.19%)) and thalamus (25 (8.83%)), which also exhibit significant numbers. Hemorrhagic strokes are less frequent but still notable in the parietal lobe (13 (12.26%)), both internal capsule and temporal lobe (12 (11.32%)), and both thalamus and caudate nucleus (11 (10.38%)). Arterial stroke is also most commonly seen in the cerebral lobes, followed by the gangliocapsular region. Venous strokes are comparatively rare, with the highest occurrences in both the parietal and temporal regions 6 (Table 6).

**Table 6:** Distribution of strokes according to type and brain region involvement, as seen in CT/MRI brain imaging n: number; %: percentage; CT: computed tomography; MRI: magnetic resonance imaging

Area involved	Ischemic stroke, n (%)	Hemorrhagic stroke, n (%)	Arterial stroke, n (%)	Venous stroke, n (%)
Pons	6 (2.12%)	1 (0.94%)	6 (2.12%)	0 (0.0%)
Midbrain	2 (0.71%)	0 (0.0%)	2 (0.71%)	0 (0.0%)
Thalamus	25 (8.83%)	11 (10.38%)	25 (8.83%)	3 (8.33%)
Lenticular nuclei	20 (7.07%)	9 (8.49%)	20 (7.07%)	1 (2.78%)
Corona radiata	19 (6.71%)	6 (5.66%)	19 (6.71%)	4 (11.11%)
Centrum semiovale	21 (7.42%)	7 (6.6%)	21 (7.42%)	2 (5.56%)
Ventricular	0 (0.0%)	4 (3.77%)	0 (0.0%)	0 (0.0%)
Corpus callosum	1 (0.35%)	2 (1.89%)	1 (0.35%)	1 (2.78%)
Internal capsule	27 (9.54%)	12 (11.32%)	27 (9.54%)	3 (8.33%)
Cerebellar	9 (3.18%)	2 (1.89%)	9 (3.18%)	2 (5.56%)
Frontal	41 (14.49%)	8 (7.55%)	41 (14.49%)	3 (8.33%)
Parietal	37 (13.07%)	13 (12.26%)	37 (13.07%)	6 (16.67%)
Temporal	26 (9.19%)	12 (11.32%)	26 (9.19%)	6 (16.67%)
Occipital	27 (9.54%)	8 (7.55%)	27 (9.54%)	4 (11.11%)
Caudate nucleus	22 (7.77%)	11 (10.38%)	22 (7.77%)	1 (2.78%)
Medulla oblongata	0 (0.0%)	0 (0.0%)	0 (0.0%)	0 (0.0%)

The analysis reveals that the most common cause of stroke, according to the TOAST classification, is UND with two or more causes, comprising 73 (73%) cases. Hyperhomocysteinemia is the leading specific cause within this category, affecting 62 (62%) cases. Large-artery atherosclerosis is the next most frequent cause, accounting for 50 (50%) cases. Cardioembolic stroke accounts for 11 (11%) cases, with four (4%) cases due to atrial fibrillation, three (3%) cases due to acute myocardial infarction, three (3%) cases due to rheumatic valvular heart disease, and one (1%) case due to infective endocarditis. Other notable causes include strokes of OTH (32 (32%)), which encompass conditions such as cerebral venous sinus thrombosis (10 (10%)), infections (9 (9%)), vasculitis (6 (6%)), and drug-induced strokes (4 (4%)). This diverse range of etiologies underscores the multifactorial nature of stroke, with both common and rare causes contributing to its incidence (Table 7).

**Table 7:** Distribution of various etiologies of stroke among study participants, based on the TOAST classification TOAST: Trial of ORG 10172 in Acute Stroke Treatment; CADASIL: cerebral autosomal dominant arteriopathy with subcortical infarcts and leukoencephalopathy; APLA: antiphospholipid antibody; n: number; %: percentage

TOAST classification	n (%)
1) Large artery atherosclerosis	30 (30%)
2) Small vessel occlusion (lacunae)	3 (3%)
3) Stroke of undetermined etiology with two or more causes	32 (32%)
4) Cardioembolic	11 (11%)
a) Acute myocardial infarction	3 (3%)
b) Atrial fibrillation	4 (4%)
c) Rheumatic valvular heart disease	3 (3%)
d) Infective endocarditis	1 (1%)
5) Stroke of other determined etiology	24 (24%)
a) Genetic polymorphism (CADASIL)	1 (1%)
b) Thrombophilic disorders (APLA, protein C deficiency)	2 (2%)
c) Vasculitis	6 (6%)
d) Moya Moya disease	1 (1%)
e) Aortic dissection	1 (1%)
f) Infection	4 (4%)
g) Hypercoagulable (pregnancy/ post-partum)	2 (2%)
h) Cerebral venous sinus thrombosis	7 (7%)

## Discussion

Stroke is a significant public health problem associated with high mortality and morbidity, severely affecting the quality of life. Globally, it is the second most common cause of death in adults. Predominantly affecting males in later years of life, the risk of stroke doubles with each decade after the age of 55. According to Donkor, factors such as diabetes mellitus, ischemic heart disease, systemic hypertension, atrial fibrillation, hyperlipidemia, prolonged alcohol use, and smoking contribute to stroke risk [2]. The prevalence of these risk factors varies across different geographical areas and populations.

In our study, the majority of participants were male (84%), with females comprising 16%. Age-related analysis showed that the risk of stroke increased with age, with 44% of patients being over 60 years old with a mean age of 56 and a standard deviation of 14.42 years. Marti-Vilalta and Arboix reported a mean age of 66.4 ± 13 years among stroke patients, with 55% aged between 65 and 84 and 33% aged between 45 and 64 [11]. Similarly, Itagi et al. found a mean age of 58.49 ± 13.97 years, with the most common age group being 61 to 70 years [12]. These findings, consistent with our study, emphasize age as a significant, non-modifiable risk factor for stroke.

Males were predominantly affected by stroke in our study, supported by findings from North and South India by Itagi et al. and Dash et al. reporting a male-to-female ratio of 2.44:1 [12,13]. Our study found a higher ratio of 5:1, potentially due to inadequate care for women and delayed hospital admission until the illness becomes severe. High rates of smoking and alcohol consumption among men, less common among women in our society, may also contribute to this disparity.

Hypertension was the most prevalent risk factor in our study, affecting 75% of patients, with a significantly higher prevalence in males (62%) compared to females (13%). Findings from studies by O’Donnell et al., Sheikh and Kulkarni, and Dash et al. align with this [8,10,13]. Smoking, observed in 51% of our stroke patients, aligns with O’Donnell et al., who found a dose-response relationship between the number of cigarettes smoked per day and stroke risk, emphasizing the importance of smoking cessation in stroke prevention programs [8].

Our study highlights the importance of managing blood pressure, lipid levels, and blood glucose for stroke prevention. Additionally, 29% of patients had a history of prior stroke, aligning with Dash et al. [13], who reported that 26% of patients had a history of previous stroke and TIA, with nearly 88% not taking medications for secondary stroke prevention. This underscores the need for aggressive primary and secondary prevention efforts, focusing on traditional modifiable risk factors.

In our study, ischemic strokes were predominant, with high occurrences in the frontal lobe (41), parietal lobe (37), occipital lobe (27), and temporal lobe (26), and significant numbers in the internal capsule (27) and thalamus (25). These findings align with those of Sheikh and Kulkarni and Jha et al., where ischemic strokes were most common, but in contrast with their reported lower prevalence of strokes in the thalamus and internal capsule [10,14]. Hemorrhagic strokes (20%) were less frequent, commonly observed in the parietal (13) and temporal lobes (12), followed by the thalamus (11) and internal capsule (12). This contrasts with Sheikh et al. and Jha et al., where hemorrhagic strokes were more prevalent in the thalamus and less common in the cortical lobes [10,14].

According to the TOAST classification, the most prevalent cause of stroke in our study was an undetermined etiology with two or more causes, accounting for 32% of cases. Nedeltchev et al. and Dash et al. reported that 33% and 57% of stroke cases, respectively, were of undetermined cause, primarily due to incomplete evaluation [10,13]. Large artery atherosclerosis accounted for 30% of stroke cases in our study, differing from Nedeltchev et al., where OTHs (30%) and cardiac embolism (24%) were more prevalent [15].

The risk factors identified in our study are consistent with findings from studies conducted in Saudi Arabia and Nalgonda. The study in the Hail region of Saudi Arabia reported hypertension (68%), diabetes mellitus (52%), dyslipidemia (47%), and smoking (34%) as major risk factors [16]. Similarly, the study from Nalgonda found hypertension (62%), smoking (49%), dyslipidemia (42%), and diabetes mellitus (33%) as prevalent risk factors [17]. These findings reinforce our results, underscoring the importance of managing these common risk factors to reduce stroke incidence.

The predominance of ischemic strokes in specific brain regions may be linked to underlying vascular pathologies such as atherosclerosis [18], which predominantly affects the large arteries supplying these areas. The higher prevalence of hypertension among stroke patients underscores the critical role of blood pressure management in stroke prevention [19]. Gender differences in stroke incidence and risk factors suggest the need for tailored prevention strategies addressing specific risk profiles [20]. The high proportion of strokes with undetermined etiology highlights the need for comprehensive diagnostic evaluations in stroke patients to identify underlying causes accurately [21]. Improved access to advanced diagnostic tools and timely medical interventions could reduce the proportion of strokes with an undetermined etiology, enabling more targeted treatments and better outcomes.

Limitations

Our study may not accurately represent the broader community's stroke cases, as it only includes patients from a single tertiary care hospital, potentially introducing selection bias. The reliance on self-reported data for some risk factors may introduce recall bias. Future studies with larger, more diverse populations and extended follow-up periods are needed to validate our findings.

## Conclusions

This study underscores the significant prevalence of hypertension, smoking, alcohol consumption, and hyperhomocysteinemia as major stroke risk factors, with ischemic strokes predominantly affecting the cerebral lobes and gangliocapsular region. Effective management of these risk factors through targeted prevention strategies, including hypertension control and lifestyle modifications, is crucial for stroke mitigation. Recognizing gender-specific differences and comorbidities, along with further research to develop tailored lifestyle interventions for at-risk patients, can enhance patient outcomes and reduce the stroke burden.
